# When a repellent becomes an attractant: harmful saponins are kairomones attracting the symbiotic Harlequin crab

**DOI:** 10.1038/srep02639

**Published:** 2013-09-12

**Authors:** Guillaume Caulier, Patrick Flammang, Pascal Gerbaux, Igor Eeckhaut

**Affiliations:** 1 University of Mons - UMONS, Biosciences Institute, Biology of Marine Organisms and Biomimetics, 7000 Mons, Belgium; 2 University of Mons - UMONS, Mass Spectrometry Research Group, Interdisciplinary Center for Mass Spectrometry (CISMa), 7000 Mons, Belgium

## Abstract

Marine organisms have developed a high diversity of chemical defences in order to avoid predators and parasites. In sea cucumbers, saponins function as repellents and many species produce these cytotoxic secondary metabolites. Nonetheless, they are colonized by numerous symbiotic organisms amongst which the Harlequin crab, *Lissocarcinus orbicularis*, is one of the most familiar in the Indo-Pacific Ocean. We here identify for the first time the nature of the molecules secreted by sea cucumbers and attracting the symbionts: saponins are the kairomones recognized by the crabs and insuring the symbiosis. The success of this symbiosis would be due to the ability that crabs showed during evolution to bypass the sea cucumber chemical defences, their repellents becoming powerful attractants. This study therefore highlights the complexity of chemical communication in the marine environment.

Chemical sensing is considered as the most ancient and the most ubiquitous mode of communication in the biosphere^1^; all living organisms are able to detect chemical cues in their environments^2^,^3^. These cues allow different types of intra- and interspecific interactions between organisms, including, for example, mate recognition, prey-predator interactions and symbiotic associations. Indeed, communication between symbionts and their hosts is needed to insure appropriate host selection and the maintenance of the symbiotic relationship through time. Chemical signals emitted by hosts are named kairomones when they elicit a commensal or a parasitic symbiosis and synomones for mutualistic ones. Symbiotic associations are very common in echinoderms^4^ and it is well known that echinoderms scent can attract various symbionts. This has been highlighted through different behavioural experiments on symbiotic polychaetes^5^,^6^,^7^, bivalves^8^, fishes^9^, crabs^10^, brittle stars^11^ and shrimps^12^,^13^. All these studies showed that odours emitted by echinoderms in surrounding seawater are detected by symbionts and trigger an attractive chemotaxy. However, the exact chemical nature of the olfactory signals involved in the selection of an echinoderm host has never been discovered. In fact, up to now, only one semiochemical involved in a marine symbiosis has been identified: the amphikuemin, a synomone secreted by some species of sea anemones and recognized by clown fishes^14^.

On the other hand, echinoderms are also well known for possessing defensive chemicals^15^,^16^ that deter predation or prevent organisms to establish on their body. These allomones, semiochemicals providing a benefit to their producers, have been described in many representatives from the five echinoderm classes, but the most studied are the noxious saponins produced by sea stars (asteroids)^17^,^18^ and sea cucumbers (holothuroids)^19^. Saponins are secondary metabolites first discovered in higher plants^20^ that are also present in several marine sponges^21^. In sea cucumbers, saponins are found in the body wall and in the viscera, including the adhesive Cuvieran tubules. Structurally, sea cucumber saponins are described as triterpene glycosides composed of an oligosaccharide chain and an aglycone based on holostane-3β-ol^15^,^22^. The carbohydrate moiety is bound to the C3 of the aglycone and may encloses up to 6 sugar units, including xylose, glucose, quinovose and 3-O-methylglucose residues^22^,^23^. Some saponins can be sulphated at the level of the sole xylose^24^ (see also Fig. 2). Because of their amphiphilic property, these molecules present deleterious membranolytic effects^25^,^26^,^27^ that make them toxic for most organisms^28^. Some saponins can be actively secreted outside the animal in the environment and may carry a warning message of the unpalatability of the holothuroid to other organisms^29^.

In this study, we hypothesized that sea cucumber saponins, even though they are harmful for most organisms, could be the kairomones specifically attracting holothuroid symbionts. To test this hypothesis, we investigated the Harlequin crab, *Lissocarcinus orbicularis*, one of the most common symbiotic organisms associated with Indo-Pacific shallow water sea cucumbers (Fig. 1). These tiny crabs are commonly observed single or by heterosexual pairs on the outer body wall, in the mouth or in the cloacum of their hosts. The symbiosis is not detrimental for the sea cucumbers although the crabs seem to be able to feed on the upper layer of the host integument. Harlequin crabs are therefore considered as commensals^30^. In the present work, chemodetection of host sea cucumbers by Harlequin crabs was demonstrated by behavioural tests in an olfactometer, an experimental device used to study the role of chemical communication in interspecific (mainly prey-predator and symbiont-host) or intraspecific interactions^5^,^31^,^32^. We used four sea cucumber species that usually host *L. orbicularis* in their natural ecosystem^30^: *Bohadschia subrubra*, *Bohadschia vitiensis*, *Holothuria scabra* and *Holothuria lessoni*. Saponins were purified from host-conditioned seawater and their attractiveness tested on crabs. Putative attractive saponin congeners were finally identified by mass spectrometry.

## Results

### Host selection is allowed by chemical communication

When both aquaria of the olfactometer were only filled with seawater, Harlequin crabs almost never moved, indicating that there was no attractive stimulus, either chemical or rheological, in the flow of regular seawater. When one aquarium was filled with seawater conditioned with individuals of one of the four holothuroids species (Table 1), the crabs first showed a typical grooming behaviour^33^ in which chemoreception organs (*i.e.* buccal appendages, antennules and legs) moved actively suggesting that they detected a chemical signal. Then, they moved into the unpaired branch up to the junction of the two paired branches where they usually stopped moving, tested the water fluxes, and significantly (*P* < 0.01) chose to orientate towards the aquarium containing sea water conditioned by their host. Over the 179 crabs that were tested with host-conditioned seawater, 151 (84%) started moving and 147 (82%) oriented correctly into the corresponding aquarium. The mean time required for *L. orbicularis* to enter into the conditioned aquarium was nearly two minutes. Most of the times, symbionts were able to adequately orientate toward the appropriate aquarium in one attempt, but 5% of the individuals first progressed in the wrong paired branch (*i.e.* towards the aquarium only filled with seawater), then stopped, went back to the intersection and finally entered in the aquarium conditioned by a holothuroid. There were no significant differences (*P* > 0.05) between males and females or between juveniles and adults. Those behavioural experiments confirm that even without any visual signal, Harlequin crabs were able to detect semiochemicals emitted by their holothuroid hosts in seawater.

### Harlequin crabs use saponins as kairomones to recognise their hosts

We then investigated whether saponins could be the chemical cues attracting Harlequin crabs to their host sea cucumbers. Saponins were purified from seawater conditioned with each of the four host species, and tested in the olfactometer. In addition, saponins were also obtained from one non-host species, *Holothuria forskali*. This species presents a geographical distribution that does not overlap with that of the Harlequin crab: *L. orbicularis* is specifically found in the Indo-Pacific area whereas *H. forskali* is restricted to the eastern Atlantic Ocean and the Mediterranean Sea^29^. For the four host species, we observed that seawater supplemented with purified saponins had the same attractive effect on crabs as seawater conditioned by living holothuroids (Table 1). Saponins from the non-host species *H. forskali* were also tested for their attractiveness towards Harlequin crabs. The results showed that these saponins were also attractive, acting as kairomones for *L. orbicularis*.

### Caracterisation of the molecular structure of saponins

**Figure 2:**
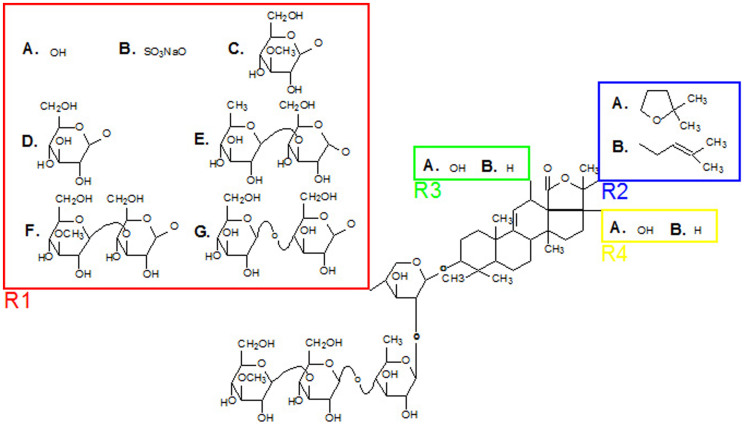
Chemical structure of saponins found in the saponin cocktails of *H. forskali*, *B. subrubra*, *B. vitiensis*, *H. scabra* and *H. lessoni*. For each saponin congener, the structure can be established using the different letters shown in Table 3.

In order to identify and characterize the molecular structures of saponins, we performed tandem mass spectrometry analyses on the purified fraction obtained from conditioned water samples and that was also used in the olfactometer experiments. Our results show that this fraction did not contain only one saponin but rather saponin cocktails consisting of a variable number of congeners ranging from 3 to 6 according to the sea cucumber species considered (Table 2, Fig. 2). Water samples conditioned by different individuals of a same species always presented the same saponin composition. A total of 14 different saponins were highlighted, differing by their sugar moiety, their aglycon moiety, and/or the presence or absence of a sulphate group (Fig. 2). In the mass spectra, no important *m*/*z* signal other than saponins was detected.[Table t3]

## Discussion

We here demonstrate that sea cucumber saponins are putative kairomones that attract symbiotic Harlequin crabs. This would involve a remarkable evolutionary mechanism in which host chemical defenses were diverted through time from their primary function. Harlequin crabs, which are permanent and obligate symbionts of sea cucumbers, survive easily on their hosts and presumably evolved adaptations to counteract the toxicity of saponins. In parallel, *L. orbicularis* developed its olfactory sense towards the detection of these molecules emitted by their sea cucumber hosts. For the four host species, it is the first time that those saponins were purified and characterized directly from the seawater surrounding the sea cucumbers. Among the 14 saponins that were detected in the different extracts, no single congener was common to all species, suggesting that Harlequin crabs recognize either only a specific structural unit common to the saponin molecules or multiple saponins. The attractiveness of *H. forskali*, a species that has never been in interaction with the symbiotic Harlequin crab in its ecosystem, is certainly due to the fact that it shares common saponins with host species (see Table 2). Although we cannot rule out that other secondary metabolites might be produced and released by sea cucumbers and used as cues for crabs, saponins are the most likely kairomone candidates at this stage.

Our results show that each sea cucumber species secretes its own cocktail of saponins which appears as a true chemical signature. It is noteworthy that even *H. scabra* and *H. lesson*i, which are phylogenetically close^34^, present different saponin signatures. Such variability in saponin cocktails could be explained by the dynamic system of evolutionary arm race, also known as Red Queen theory^35^ that presumably occur between sea cucumbers and their predators. In this hypothesis, holothuroids would have developed a chemical defence system based on saponins which would considerably reduce their predation. On the other hand, specialised predators would need to develop several strategies to overcome the noxious effect of saponins. Saponin diversification would thus be an important evolutionary pressure on sea cucumbers to efficiently deter predators.

We suggest that Harlequin crabs can also take advantage from the sea cucumber chemical defence for protecting themselves from their own predators. Similar hypotheses were demonstrated in different studies which highlighted that several species of amphipods are associated with specific algae and pteropods in order to benefit from their chemical defences^36^,^37^,^38^. To settle on sea cucumbers would provide to the Harlequin crab an utmost evolutionary advantage that would explain its successful Indo-Pacific distribution: the crabs became protected from predators thanks to their host's chemical defence.

## Methods

### Sampling procedure

One hundred and sixty different Harlequin crabs *Lissocarcinus orbicularis* (Dana, 1852), both males and females or juveniles and adults, and thirty-seven sea cucumbers from five different species were sampled in the lagoon of the coral reef of Toliara (South-West of Madagascar) and used for the behavioural tests. Within the five sea cucumber species, four are recognized hosts of the Harlequin crab^30^ (*Holothuria scabra* (Jaeger, 1833) (*N* = 8), *Holothuria lessoni* (Massin, 2009) (*N* = 8), *Bohadschia subrubra* (Quoy & Gaimard, 1834) (*N* = 7) and *Bohadschia vitiensis* (Semper, 1968) (*N* = 11)) whereas the last species, *Holothuria forskali* (Delle Chiage, 1823) (*N* = 3), presents a geographical distribution that does not overlap the distribution of the Harlequin crab. Sea cucumbers were sampled by scuba diving or were hand collected at low tide around the coral reef of Toliara (Madagascar) or in Banyuls-sur-mer (France). Olfactometry measurements were conducted at the “Institut Halieutique et des Sciences Marines” (Toliara, Madagascar). Saponin extraction from host-conditioned seawater (see below) was performed at the same Institute. Saponin extraction from *H. forskali*-conditioned seawater and mass spectrometry analyses were done at the University of Mons (Belgium). Animals used in our experiments were maintained and treated in compliance with the guidelines specified by the Belgian Ministry of Trade and Agriculture.

### Behavioural experiments

The Davenport olfactometer used in this study was a Y-shaped glass tube of 20 cm long and 10 cm of section whose paired branches are connected to two aquaria of 5 l in volume. One of the aquaria contained test seawater and the other was filled with control seawater. Test water consisted of seawater conditioned with living sea cucumbers or supplemented with purified saponins (see below). For the latter, a purified saponin solution was introduced into one of the two aquaria of the olfactometer at a flow rate of 10 ml/min using a peristaltic pump (Amersham P-1). Water flowed from the two aquaria through the olfactometer and was evacuated at the base of the unpaired branch of the Y-tube. Flow turbulence inside the olfactometer was controlled using fluoresceine before experiments, and the water flux was regulated to a speed of 2–3 cm/s. Water used for these experiments was filtered natural seawater, 26°C and 35‰ salinity, pumped directly in the sea.

During a typical trial, one Harlequin crab was introduced at the base of the unpaired branch of the Y tube. If it was not stimulated, the crab remained at the base of the branch without moving and the run was aborted and considered as null after 10 min. If the crab was stimulated, it moved into the unpaired branch up to the junction of the two paired branches and potentially chose to orientate itself into one of the paired branches. Two types of behaviours were therefore recorded in the Y tube: the motion behaviour, when crabs moved for at least 10 cm into the unpaired branch towards the stimulation source, and the orientation behaviour, when crabs entered one of the two paired branches and reached the corresponding aquarium. In this case, the trial was stopped since crabs had reached the end of the olfactometer. To test the significance of the motion behaviour, the number of times crabs started to move under a chemical stimulation (*i.e.* chemotaxy) was compared by a chi-squared test to the number of times crabs were moving when control seawater filled both aquaria. The orientation behaviour was tested by comparing the percentage of crabs that entered the aquarium A to a random distribution (50/50). Statistical tests were realized with the R software. At least twenty different crabs were tested with each species of sea cucumbers or saponin extracts, and aquaria A and B were exchanged every five runs. The entire olfactometer was washed between each trial in order to remove remaining sea cucumber scents or saponins.

### Water conditioning and saponin extraction

Sea cucumber-conditioned water was prepared by incubating one individual (total weight of 300 to 600 g) for 20 minutes in 5 l of filtered natural seawater or, alternatively, for 4 hours in 1 l of artificial seawater (made by dissolving 26 g of NaCl, 12 g MgCl_2_, 0.7 g of KCl, 1.47 g of CaCl_2_ and 0.2 g of NaHCO_3_ in MQ water). The former was used directly as host test water in the olfactometer while the latter was used for saponin purification. For behavioural experiments, we used a minimum of 7 conditioned seawater replicates per species, each prepared with a different individual. For saponin purifications, a minimum of 3 replicates were used.

Saponin purification was performed by passing seawater conditioned by the four host-species (*B. subrubra*, *B. vitiensis*, *H. scabra* and *H. lessoni*) and by the non-host species *H. forskali* through a chromatography column filled with Amberlite Xad-4 resin (Sigma-Aldrich, St. Louis, MO)^29^. The column was washed with ultrapure water to eliminate salts and the saponins were eluted with 40 ml of methanol. The methanolic elution was then evaporated to dryness and the extract was dissolved in 20 ml water in order to undergo a liquid-liquid extraction against isobutanol (1:1). The butanolic fraction, containing the purified saponins, was either stored for subsequent mass spectrometry analyses or evaporated to dryness, dissolved in seawater and used for behavioural experiments. In this case, a concentration of 200 mg/l was used, corresponding approximately to the quantity of saponins detected in sea cucumber-conditioned seawater^29^.

### Mass spectrometry analyses

Saponins released in seawater by the five sea cucumber species were analyzed with a Waters QToF Premier tandem mass spectrometer using Matrix-assisted Laser Desorption/Ionization for the ion production (positive ion mode). The MALDI source was constituted by a nitrogen laser, operating at 337 nm with a maximum output of 500 mW delivered to the sample in 4 ns pulses at 20 Hz repeating rate. All samples were prepared using a 10 mg/ml solution of α-cyano-4-hydroxycinnamic acid (α-cyano) in acetone as the matrix. The matrix solution (1 μl) was spotted onto a stainless steel target and air dried. Then, 1 μl of each butanolic fraction was applied onto the spots of matrix crystals, and air dried. Finally, 1 μl droplets of a solution of NaI (2 mg/ml in acetonitrile) were added to the spots on the target plate. Because of the high affinity of alkali cations for triterpene glycosides^39^, the mass spectra of all the saponins are dominated by [M + Na]^+^ ions. For the MALDI MSMS experiments, the ions of interest were mass-selected by the quadrupole mass filter. The selected ions are then submitted to collision against argon in the T-wave collision cell (pressure estimated at 0.9–1 mbar) and the laboratory frame kinetic energy was selected to afford intense enough product ion signals. All the ions exiting the collision cell, either the product ions or the non dissociated precursor ions, were finally mass measured with the oa-ToF analyzer. Time-of-flight mass analyses were performed in the reflectron mode at a resolution of about 10 000. Mass spectra were analysed with MassLynx 4.1 software, chemical structures were determined from fragmentation schemes calculated on tandem mass spectra and from literature. Saponins molecular structures were finally drawn using ChemWindow 6.0.

## Figures and Tables

**Figure 1:**
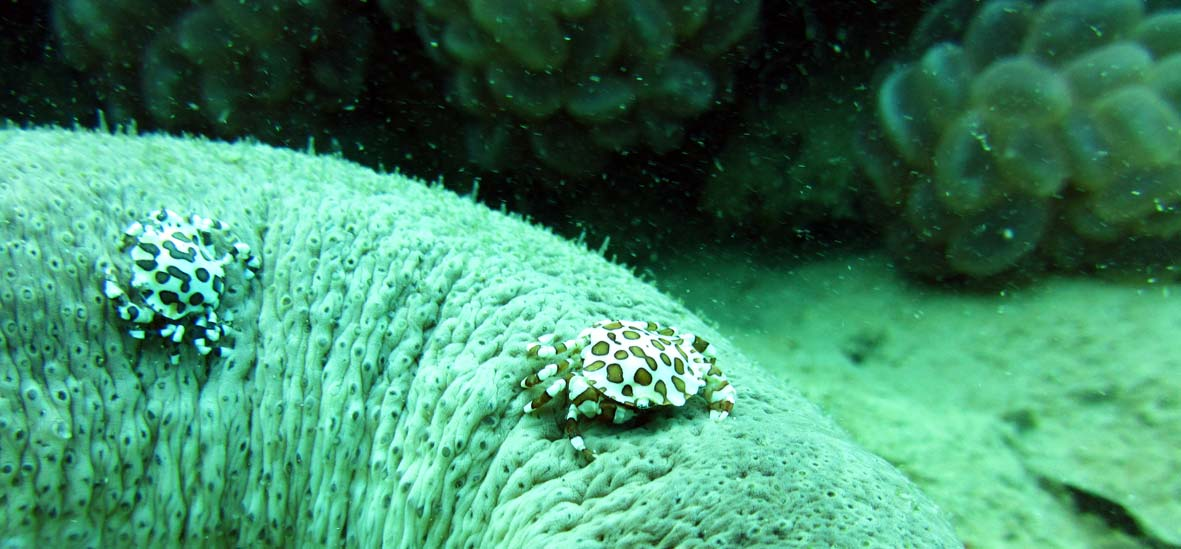
Harlequin crabs *Lissocarcinus orbicularis* on their sea cucumber host (*Bohadschia vitiensis*).

**Table 1:** Results of behavioural experiments using the olfactometer. 10 different tests were realised using a minimum of 20 different Harlequin crabs. At least 7 individuals from each holothuroid species were used to prepare conditioned water, and at least 3 individuals to purify saponins. Crab behaviour observation was limited to 10 minutes. Results show the number of times crab remained stationary in the impaired branch (Null), orientate adequately toward the stimulus source (A), orientate toward the control aquarium (B). The P-value of the chi^2^ test and the binomial test are respectively found in motion and orientation behaviour columns. Significant results (P-value < 0.01) are in bold

		Results				Statistics	
Aquarium A Vs Aquarium B		Trials	Null	A	B	Motion behaviour	Orientation behaviour
**Negative control**							
1	seawater Vs seawater	80	73	3	4	/	1
**Water conditioned by host sea cucumbers**							
2	*Bohadschia vitiensis* Vs seawater	119	18	98	3	**2.2 10^−16^**	**2.2 10^−16^**
3	*Bohadschia subrubra* Vs seawater	20	4	16	0	**3.6 10^−5^**	**3.1 10^−5^**
4	*Holothuria scabra* Vs seawater	20	5	15	0	**1.2 10^−4^**	**6.1 10^−5^**
5	*Holothuria lessoni* Vs seawater	20	2	18	0	**2.1 10^−6^**	**7.6 10^−6^**
**Water conditioned by saponins extracted from host sea cucumbers**							
6	Saponins *B. vitiensis* Vs seawater	20	5	15	0	**1.2 10^−4^**	**6.1 10^−5^**
7	Saponins *B. subrubra* Vs Seawater	20	4	16	0	**3.6 10^−5^**	**3.1 10^−5^**
8	Saponins *H. scabra* Vs seawater	20	3	17	0	**9.3 10^−6^**	**1.5 10^−5^**
9	Saponins *H. lessoni* Vs seawater	20	2	18	0	**2.1 10^−6^**	**7.6 10^−6^**
**Water conditioned by saponins extracted from a non-host sea cucumber**							
10	Saponins *H. forskali* Vs seawater	20	4	15	1	**3.6 10^−5^**	**5.1 10^−4^**

**Table 2:** Chemical characterisation of the saponins signatures contained in the water conditioned by the five species of sea cucumbers tested in behavioural experiments. “X” means that the saponin congener was found in the cocktail of the corresponding species. [M + Na]^+^ represents sodium-cationized saponins, *i.e.* saponin ions, produced by MALDI and detected by mass spectrometry at the corresponding mass-to-charge ratio (*m*/*z*). Corresponding chemical structures can be found in Fig. 2

Saponins	[M + Na]^+^	*H. forskali*	*B. subrubra*	*B. vitiensis*	*H. scabra*	*H. lessoni*
Bivitoside B	1111			X		
Holothurinoside C	1125	X			X	
Desholothurin A	1141	X				X
Scabraside A	1227				X	
Holothurin A2	1229				X	X
Scabraside B	1243				X	X
Holothurinoside M	1301	X				
Holothurinoside A	1303			X		
Holothurinoside N	1317	X				
Holothurinoside F	1433	X		X		
Bivitoside C	1434		X			
Holothurinoside G	1449	X	X	X		
Holothurinoside H	1463		X	X		X
Arguside C	1465			X		

**Table 3 t3:** Table related to Figure 2

Saponins	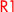	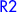	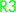	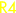
**Holothurinoside C**	**A.**	**A.**	**B.**	**B.**
**Desholothurin A**	**A.**	**A.**	**A.**	**A.**
**Bivittoside B**	**A.**	**B.**	**A.**	**B.**
**Scabraside A**	**B.**	**A.**	**A.**	**B.**
**Scabraside B**	**B.**	**A.**	**A.**	**A.**
**Holothurin A2**	**B.**	**B.**	**A.**	**A.**
**Holothurinoside M**	**C.**	**A.**	**A.**	**B.**
**Holothurinoside N**	**C.**	**A.**	**A.**	**A.**
**Holothurinoside A**	**D.**	**A.**	**A.**	**A.**
**Holothurinoside F**	**E.**	**A.**	**A.**	**B.**
**Holothurinoside G**	**E.**	**A.**	**A.**	**A.**
**Bivittoside C**	**F.**	**B.**	**A.**	**A.**
**Holothurinoside H**	**F.**	**A.**	**A.**	**B.**
**Arguside C**	**G.**	**A.**	**A.**	**A.**
